# Spatial Relationship between Heat Illness Incidence and Heat Vulnerability in Gurye and Sunchang, South Korea, 2018

**DOI:** 10.3390/ijerph20115992

**Published:** 2023-05-29

**Authors:** Chaeyeon Yi, Hyukgi Kwon

**Affiliations:** Research Center for Atmospheric Environment, Global Campus, Hankuk University of Foreign Studies, Yongin 17035, Republic of Korea

**Keywords:** Korea, heatwave damage, heatwave vulnerability, heatwave risk, heat-related illnesses

## Abstract

Heatwaves, along with their affiliated illnesses and mortalities, are increasing in frequency and severity under climate change. Spatial analyses at the level of census output areas can produce detailed maps of heatwave risk factors and potential correlated damages, thus contributing to practical policies to reduce the risk of heatwave illnesses. This study analyzed the 2018 summer heatwave in Gurye and Sunchang counties in South Korea. To compare damages and analyze the detailed causes of heatwave vulnerability, spatial autocorrelation analyses were conducted, incorporating weather, environmental, personal, and disease factors. Gurye and Sunchang, although similar in demographics and region, exhibited large differences in heatwave damage specifically in the number of heat-related illness cases. In addition, exposure data were constructed at the census output area level by calculating the shadow pattern, sky view factor, and mean radiant temperature, revealing a higher risk in Sunchang. Spatial autocorrelation analyses revealed that the factors most highly correlated with heatwave damage were hazard factors, in the case of Gurye, and vulnerability factors, in the case of Sunchang. Accordingly, it was concluded that regional vulnerability factors were better distinguished at the finer scale of the census output area and when detailed and diversified weather factors were incorporated.

## 1. Introduction

With the growing interest in climate change and extreme weather, researchers have explored the response and adaptation to climate change, with particular emphasis on vulnerable areas. With global warming increasing, heatwaves have also been intensifying, causing many heat-related illnesses and deaths. From 1973 to 2019, the daily maximum temperature and number of heatwave days (daily maximum temperature > 33 °C, definition of heat wave by the Korea Meteorological Administration by 2020) increased by 1.5 °C and 6.9 days, respectively. In 2018, when heatwaves were designated as natural disasters, 31.5 heatwave days were recorded, three times greater than the yearly average (10.5) from 1986 to 2017 [1]. Specifically, the average numbers of heatwave-induced illnesses and deaths per year between 2011 and 2017 were 1132 and 11, respectively, and by 2018 these values had considerably increased to 4526 and 48, respectively [2]. Climate change is expected to further exacerbate the severity of heatwaves, as it is expected that the global average temperature will increase by 1.9–5.2 °C by the end of the 21st century [3], with the annual frequency of heatwaves increasing two- to seven-fold by 2050 [4]. Such an increase in heatwave frequency and intensity will cause inevitable damage to society, including the loss of human lives and health, and economic losses. For example, human casualties caused by heatwaves and droughts accounted for 91.6% of weather-related disasters from the late 1980s to 2003 in the US, and an estimated 35,000 deaths and over USD 13 billion in damage occurred in Europe in 2003 during severe heatwaves [5]. In particular, when the daily maximum temperatures exceed 33 °C, the mortality rate tends to sharply increase [6]. Heatwaves cause heat-related illnesses, such as allergies, asthma, exhaustion, fainting, and dehydration [6]; they also increase the incidence of various diseases by weakening the body’s thermoregulatory function [7]. Numerous studies have related heatwave damage to vulnerable populations; for example, in an Australian study, it was reported that a considerable number of elderly persons died as a result of heatwaves compared with other age groups [8]. Further, because there are differences in the incidence of heat-related illnesses between rural and urban areas, it has also been shown that heatwave damages differ by global region [9]. From a regional perspective, heatwave-related damage occurs disproportionately, even in regions belonging to a single administrative space [10]. To achieve more effective adaptation and response to heatwaves, comprehensive approaches considering regional characteristics in various fields are required [11].

In Dhaka, the capital of Bangladesh, a study was conducted to analyze the mitigation of urban heat island effects and the maintenance of thermal comfort using the heat vulnerability index, along with the spatial distribution of urban heat island risk [12]. In recent studies of heatwave vulnerability (exposure, sensitivity, and adaptive capacity), the focus has been on heatwave exposure and adaptive capacity components; however, heatwave vulnerability has a complex relationship with geographic and climatic factors, spatial and temporal factors, and socioeconomic factors [13].

In particular, heatwave mortalities occur more frequently among persons aged ≥65 years or in vulnerable classes who lack the economic capability to respond [6]. Regionally, each location is associated with distinct damage risks, even in the same administrative district, due to the large difference in radiant temperature in summer [14]; therefore, a spatial analysis of damage risks is required. According to the Fifth Report of the Intergovernmental Panel on Climate Change (IPCC), heatwave “risk” refers to the degree of impact caused by the interaction among hazard, exposure, and vulnerability, on which the heatwave risk index is based. As the spatial resolution of the index is limited to cities, counties, and districts, identifying the detailed characteristics of regions where patients with heat-related illnesses actually occur, and directly reflecting them in metrics recognized by local governments, remains challenging. For example, Choi et al. [15] constructed statistical data on the degree of exposure, sensitivity, and adaptive capacity to climate change of administrative neighborhoods in Seoul, while analyzing the spatial distribution of heatwave vulnerability. The authors found that the vulnerability index of each administrative neighborhood can serve as important data for establishing heatwave adaptation policies. However, such data are not available for all districts in South Korea, unlike Seoul, which has well-constructed statistical data and excellent administrative power, and no analyses have been conducted on regions smaller than districts. Bae et al. [14] created a heatwave map for census output areas in Cheongju City and analyzed the spatial relationship between heatwave exposure risk levels and the inhabitability of neighborhoods in which vulnerable populations reside, ultimately revealing high vulnerability in the old city center. Spatial analyses at the level of census output areas can produce a detailed map of heatwave risk factors and potential correlated damages, and contribute to establishing practical policies to reduce the heatwave risk. However, owing to limitations in data collection, variables for factors related to more chronic disease in vulnerable persons were omitted.

In the present study, the county of Gurye, in Jeollanam-do Province, and Sunchang, in Jeollabuk-do, which have similar populations and geographical environments, were selected as study areas in which to investigate the severe heatwave of the summer of 2018. To compare regional heatwave damages and analyze the detailed causes of heatwave vulnerability at the level of census output areas, spatial autocorrelation analyses were conducted, incorporating weather, environmental, personal, and disease factors. Heatwave damage risk maps were prepared using the vulnerability and heat exposure information obtained from the spatial autocorrelation analyses, and the detailed causes of heatwave vulnerability at the level of census output areas were derived through a comparison of the number of patients with heatwave-related illnesses. The results are useful for the establishment of detailed heatwave response policies for local governments.

## 2. Materials and Methods

### 2.1. Heatwave Damage Status Data

In this study, the heat-related illness data collected by the Korea Disease Control and Prevention Agency (KDCA) and the customized research database of the National Health Insurance Service (NHIS) were used to analyze the heatwave damage status in Gurye and Sunchang in 2018. The KDCA data reflect the number of illnesses and deaths caused by heatwaves, as documented by emergency room admissions in the summer of 2018, and are reported by 519 institutions across the country. These data include the date and time of symptoms; the gender and age of the patient; and the city, county, and district. Because these data are limited to patients with heat-related illnesses who visited emergency rooms (rather than outpatient visits), the total count may underrepresent the total number of heat-related illnesses that actually occurred (Table 1). The heat-related illness surveillance system follows the scope and definition of the Korean Standard Classification of Diseases (KCD)-8 (Table 2). Data were collected based on the ‘locations of emergency rooms’ and the ‘areas of symptoms’ from 2018. The customized research data provided by the NHIS were derived from insurance claim amount information for all patients enrolled in medical insurance, and the health information collected, stored, and managed by the NHIS was provided after processing to be used for policy and research [9].

This study protocol was reviewed and approved by the Institutional Review Board (IRB) of the Ministry of Health and Welfare of South Korea. As this was an observational study without intervention and de-identified statistical data were used, the requirement for informed consent was waived by the same IRB committee. All methods were carried out in accordance with the Korean government’s guidelines for health and medical data utilization.

In 2018, there were 4526 cases of heat-related illnesses and 48 deaths reported by the KDCA heat-related illness surveillance system. However, according to customized research data from the NHIS, the number of heat-related illnesses was much higher at 44,094, approximately 10 times more than what was reported by the KDCA. Therefore, we analyzed the characteristics of the data sets from each institution.

### 2.2. Target Sites and Weather Factor Data

Baek et al. analyzed correlations using heatwave influence variables, such as sensitivity, adaptive capacity, and exposure in 229 local governments across South Korea, as well as patient data with heat-related illnesses from the KDCA in 2018 [16]. The authors used SPSS to analyze the correlations between 25 influential variables and the number of patients with heat-related illnesses, revealing 11 significant factors. Accordingly, the authors calculated the 11-dimensional Euclidean distance for 229 × 229 local government pairs and selected Gurye and Sunchang as the most similar control groups.

**Figure 1:**
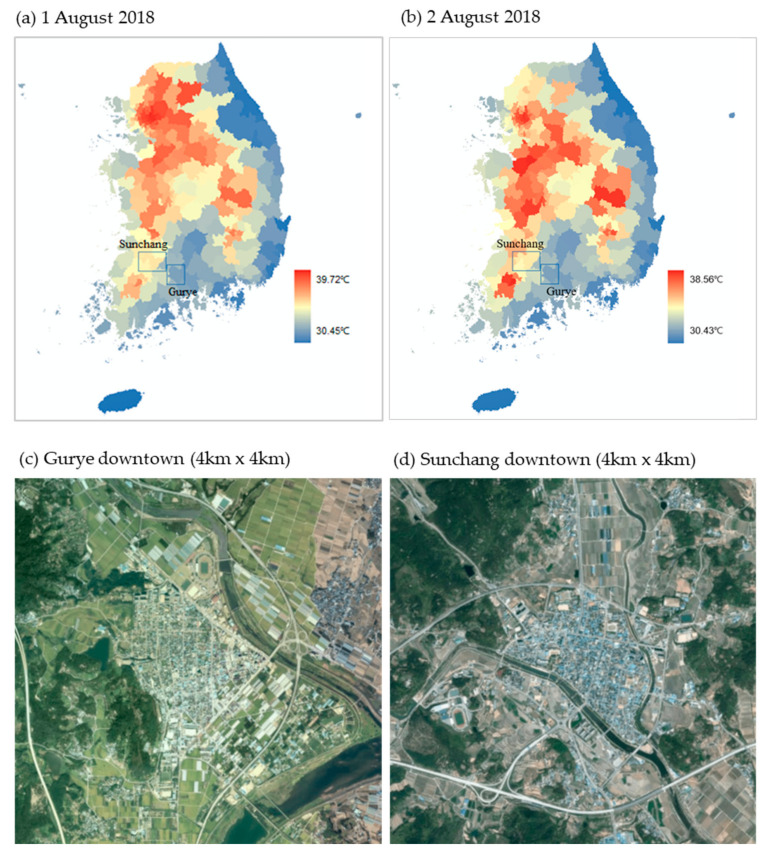
Distribution of maximum temperature (T_MAX_) in cities, counties, and districts (1–2 August 2018 (**a**,**b**)) and study areas (**c**,**d**).

In the present study, detailed spatial distribution data were used to analyze the weather status of heatwave days for the target regions. Automatic weather station (AWS) units are operated by KMA. In addition, we utilized point data representing the surrounding environment by selecting suitable locations based on the Weather Observation Standardization Act; however, these points are not always adjacent to major residential areas. Thus, grid-based temperature data that allow detailed analysis of neighborhood weather forecasts of KMA were used. Yi et al. [17] used the KMA neighborhood weather forecast data (5 km resolution) and the Gaussian process regression model (GPRM) to predict the impact of heatwaves and calculated detailed weather data with a 1 km resolution by interpolating sub-variables, such as the altitude above the sea level, inclination angle, distance from the shoreline, land cover, depth of depressed topography, east–west slope, north–south slope, and slope direction. The detailed weather data generated through GPRM are suitable for the analysis of the impacts of heatwaves on pedestrian environments, as they yield the daily maximum and minimum temperatures with higher accuracy than those of the neighborhood weather forecasts in dry urban areas and farmlands. In the present study, the number of heatwave days, duration of heatwave, and number of tropical nights in Gurye and Sunchang in the summer of 2018 were calculated, compared, and analyzed using the GRPM detailed weather field data (Figure 1a,b).

### 2.3. Environmental, Personal, and Disease Factor Data at the Level of Census Output Area

Data at the level of census output area were constructed to analyze the detailed causes of heatwave vulnerability in Gurye and Sunchang (Figure 1c,d). A census output area is the minimum statistical area constructed based on the basic unit district considering the population scale (optimal 500 persons), socioeconomic homogeneity (housing type and land price), and area geometry (statistical geographic information service, SGIS, of Statistics Korea, https://sgis.kostat.go.kr/ (accessed on 21 May 2023)). The size of the census output area corresponds to approximately 1/30th of Eup-Myeon-Dong, which are small administrative districts in Korea, and is the minimum statistical area for which Statistics Korea provides information. There are 50 census output areas in Gurye and 58 in Sunchang. The statistical data at the level of census output areas include the infant population; elderly population; population density; average age; aging areas; old-age dependency ratio; number of old houses; and agricultural, forestry, and fishery populations.

To analyze the detailed causes of heatwave vulnerability, data that can be constructed for each census output area were collected. According to the “Annual Report on the Status of the Heat-Related Illnesses caused by Heatwaves in 2018”, published by the KDCA, the most common location of heat-related illnesses was at ‘home’, comprising approximately 13.8% of the total and approximately 51.9% of indoor places. In the case of elderly people (≥65 yr) in Seoul, 41.2% of heat-related illnesses over the last five years occurred at ‘home’. In particular, old houses are affected by building insulation, which can reduce the change in indoor temperature in response to the external environment dictated by meteorological events [18]. In Korea, insulation standards were legally stipulated for the first time in the “Enforcement Decree of the Building Act” of September 1979. In the present study, the proportion of old houses by census output area, developed by the National Disaster Management Research Institute, was applied and the map of this information indicated the degree of aging for each census output area with respect to the Korea Safety Map on the facility safety of old buildings via grades 1–10.

In addition, the distribution status of major diseases among health insurance subscribers from the NHIS was used to reflect the number of patients with underlying diseases who are particularly vulnerable to heat-related illnesses. The major diseases applied were hypertension (disease codes: I10–I15), diabetes (E10–E14), hyperlipidemia (E78), cancer (number of cancer patients registered for extended health insurance—V193), cardiac infarction (I21, I22), and stroke (I60–I64). The number of patients with underlying diseases by census output area in Gurye and Sunchang was constructed per 200 m grid using the distribution of the number of patients and then reflected in the spatial correlation analysis.

### 2.4. Method to Calculate Perceived Heat Exposure Data

**Figure 2:**
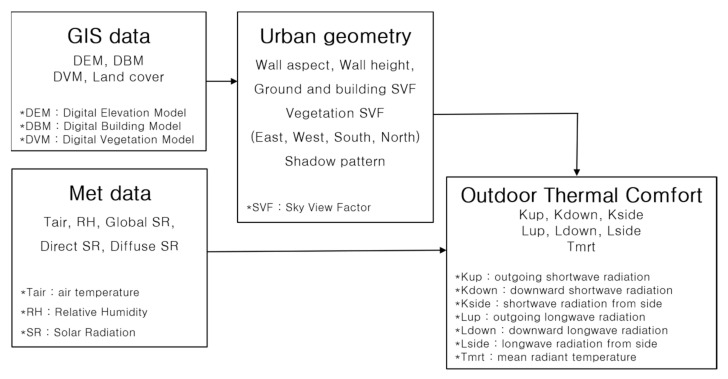
Flow chart of SOLWEIG model. * indicates the name and description of the dataset.

**Figure 3:**
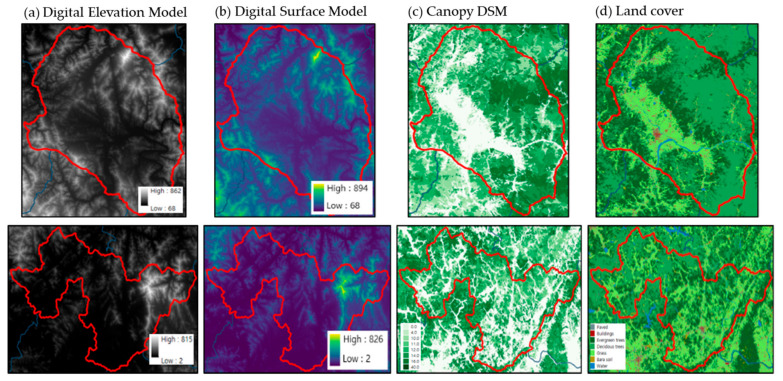
Input data shown on the following: (**a**) digital elevation model, (**b**) digital surface model (building and ground), (**c**) canopy digital surface model, and (**d**) land cover in Gurye (above), and Sunchang (below).

The solar radiation SOlar and LongWave Environmental Irradiance Geometry model (SOLWEIG) was used to analyze the heat exposure status in the target regions, as developed by the Urban Climate Group at the University of Gothenburg in Sweden [19]. Version 1.0 was released in 2009, and can be used to calculate the shading information as well as the sky view factor (SVF) for each grid using detailed topography, building height, and land cover information. With SOLWEIG, one can also model spatiotemporal changes in three-dimensional radiation flux and mean radiant temperature, which are important for heat vulnerability assessment (Figure 2). In this study, the SOLWEIG 2019a version was used, which can apply parameters according to land cover. Ref. [20] confirmed that the SOLWEIG model accurately simulated the actual radiation flux through observational experiments, whereas Ref. [21] verified SOLWEIG for summer and winter, as well as clear and cloudy days in the Jungnang area of Seoul, in addition to observing high performances (R^2^ = 0.98) for the upward longwave radiation and an RMSE of 25.84 W·m^−2^. To use SOLWEIG, information on detailed topography, building height, land cover, and vegetation height is required. In this study, the terrain height was calculated using a digital elevation model from the National Geographic Information Institute (published in 2013), and the building height was calculated using the road name–address–building map (published in February 2021) from the Ministry of the Interior and Safety (MOIS). In addition, land cover was calculated using a land cover classification map of MOE (published in 2019) and vegetation height information was calculated using the stock map (1:5000) of the Korea Forest Service (published in July 2019) to construct 5 m resolution SOLWEIG input data for Gurye and Sunchang (Figure 3).

**Figure 4:**
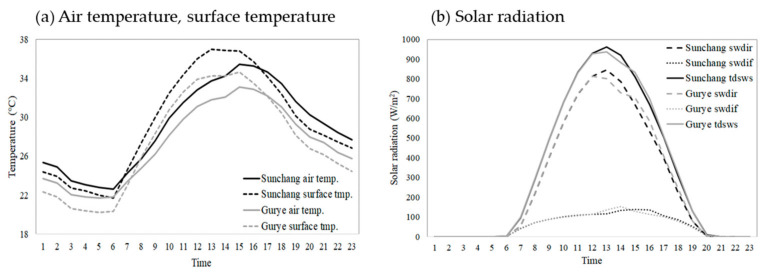
Time series distribution of meteorological data extracted from LDAPS (1 August 2018).

For SOLWEIG simulation, hourly weather factors such as temperature, humidity, and solar radiation (global/direct/diffuse solar radiation) in the target regions were required. For the KMA observation data, solar radiation was observed only at Automated Synoptic Observing System (ASOS) locations. As there are no ASOS locations in Gurye, and applying the values from a single location has limitations when reflecting detailed regional characteristics, the hourly temperature, humidity, and solar radiation (global/direct/diffuse solar radiation) data of the local forecast model (LDAPS, Unified Model) operated by the KMA were used in this study. LDAPS data maintain a spatial resolution of 1.5 km and consist of 70 layers up to approximately 40 km vertically. Specifically, hourly temperature, humidity, global solar radiation (*tdsws*), direct solar radiation (*swdir*), and diffuse solar radiation (*swdif*) were extracted according to the corresponding grids of Gurye and Sunchang, and converted to ASCII. The largest number of heat-related deaths occurred on 1 August 2018; thus, it was selected as the case date. When the weather data were extracted, it was found that the overall temperature was higher in Sunchang (a comparative average maximum temperature of +2.4 °C, 16:00 to 18:00) and the surface temperature was also slightly higher in Sunchang than in Gurye. Solar radiation was slightly higher in Sunchang after 13:00, and the largest difference of 38.2 W·m^−2^ was observed at 14:00 with similar daily patterns (Figure 4), although these differences were not significant.

### 2.5. Spatial Autocorrelation Analysis Method

To conduct a more comprehensive analysis of the underlying causes of heatwave damage, we classified vulnerability factors into four categories: weather, environmental, personal, and disease (Table 3). Vulnerability to heatwaves is higher in areas where more susceptible classes are likely to live.

To visualize the spatial distribution of each heatwave damage vulnerability factor, and test for autocorrelation, seven stages were applied based on the Jenks natural breaks classification method. This method reduces the variance within a grade based on the average of all values, maximizes the variance between each grade, and is mainly used for dividing 7–10 classes [22]. Data in the state maps are categorized using a modification of the Jenks natural breaks classification method. The Jenks method clusters data into groups that minimizes the within-group variance and maximizes the between-group variance. The modification rounds the data cut-off points to assist map reading by a general audience [23].

In the spatial analysis, Global Moran’s *I* in ArcGIS v.10.3 was used. Specifically, Moran’s *I* indicates whether the spatial arrangement of factors is purely coincidental or whether areas with similar variances spatially form a series of patterns for a specific phenomenon [24]; Equation (1):

(1) $$I=\frac{n}{\sum_{j}\sum_{i}}\times \frac{\sum_{i}\sum_{j}\omega_{ij\;\left(x_{i}-\bar{x}\right)\;\left(x_{j}-\bar{x}\right)}}{\sum_{i}{\left(x_{i}-\bar{x}\right)}^{2}}$$

where the product of the deviation is calculated from the overall mean of the target variable in region *i* (*x_i_*) and the neighboring variable in region *j* (*x_j_*); *n* is the number of census output areas; and *w_ij_* is the weight that constitutes the spatial weight matrix. Moran’s Ⅰ coefficient maintains a positive value if the adjacent region has similar characteristics, thereby indicating a positive spatial autocorrelation, whereas the value is negative if the adjacent region has different characteristics, indicating a completely negative spatial autocorrelation. Spatial autocorrelation factors were extracted accordingly for the heatwave damage vulnerability factors.

Hierarchy analysis is a method of constructing and analyzing complex decisions using mathematics and psychology. It was developed by Thomas L. Saaty in the 1970s [25] and recognizes that if the goals or evaluation criteria of decision-making are multiple and complex, they are hierarchized. Thus, the approach decomposes the main factors into the detailed sub-factors and calculates the importance through pairwise comparison of these sub-factors [26]. Using the heat exposure risk weights derived through hierarchy analysis, heat exposure based on mean radiant temperature representing human heat vulnerability and risk was overlapped to identify areas with high risk of heatwaves and vulnerable areas, and their characteristics were analyzed.

## 3. Results and Discussion

### 3.1. Number of Heat-Related Illnesses and Heatwave Risk Characteristics in Gurye and Sunchang

A comparison was made between the number of patients with heat-related illnesses in the KDCA and NHIS data sets. The KDCA data set, collected from emergency rooms, reported sixteen patients in Gurye and three in Sunchang. In contrast, the NHIS data set, collected from all medical institutions, showed 27 patients in Gurye and 152 patients in Sunchang. Ultimately, the number of patients with heat-related illnesses was substantially different depending on the counting method of each institution.

In the case of both Gurye and Sunchang, detailed indicators corresponding to hazard, exposure, and vulnerability were calculated and compared according to the heatwave risk criteria suggested by the IPCC (Table 4). Specifically, hazard indicates the severity of climate change, and its subsequent physical impact through the daily maximum temperature; the number of days when the daily maximum temperature ≥ 33 °C; and the relative humidity. Here, hazard was calculated using the ASOS data of KMA. In Gurye, which notably has no ASOS locations, the data from the closest Suncheon location were used. Exposure represents the degree to which humans or objects are spatially or environmentally subjected to climate-change-based damages and specifically pertains to persons ≥ 65 years, the elderly living alone, and recipients of basic livelihood security. Vulnerability is an indicator of the degree of sensitivity to climate change damage or the degree of insufficient response capacity, and involves the urbanized area ratio, financial independence, as well as the number of emergency medical institutions per unit population. The resident registration demographic data of MOIS (https://jumin.mois.go.kr/ (accessed on 21 May 2023)) were used for the population, whereas the data provided in the National Statistical Portal of Statistics Korea (https://kosis.kr/ (accessed on 21 May 2023)) were used for statistical information, such as the elderly living alone, basic livelihood security recipients, urbanized area ratio, and green area ratio. The weight calculated through analytic hierarchy process (AHP) by the Ministry of Environment (MOE) was 0.37 for hazard, 0.36 for exposure, and 0.27 for vulnerability; thus, the hazard index produced the highest weight. Comparatively, when the detailed heatwave risk indicators of MOE were compared, there was no significant difference in hazard index between Sunchang and Gurye, even though the daily maximum temperature, number of heatwave days, and number of tropical nights were slightly larger, while relative humidity was approximately 7% lower in Sunchang. Similarly, no significant difference in exposure was observed for most factors. With respect to vulnerability, the green area ratio was approximately 15% higher in Sunchang, whereas all remaining factors were similar across both locations.

### 3.2. Weather Factors in the Census Output Areas in 2018

**Figure 5:**
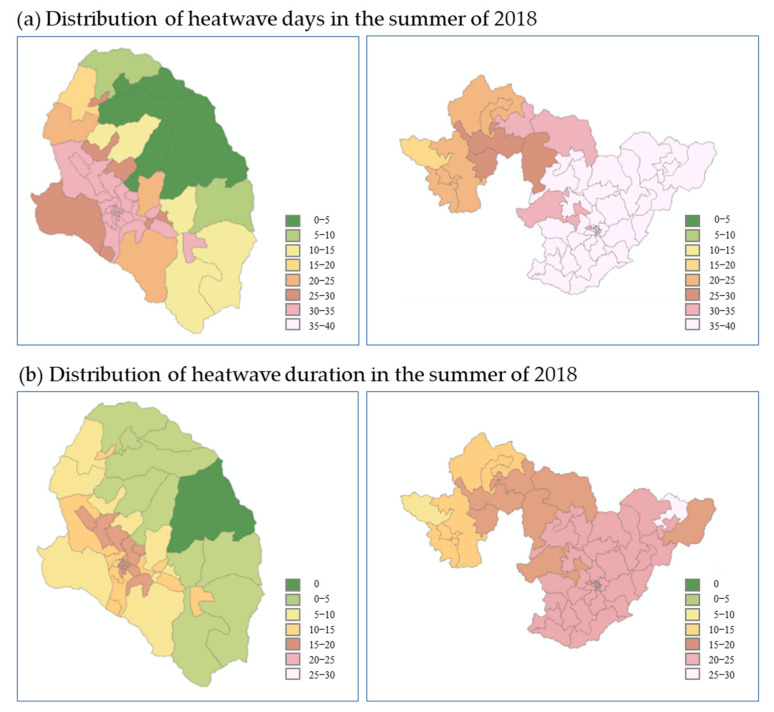
Number of heatwave days during the summer of 2018 in Gurye and Sunchang.

To compare the weather factor characteristics across the two regions, the number of heatwave days, duration of heatwave, and number of tropical nights at the level of census output areas were compared for 92 days in the summer of 2018 (June–August) using GPRM. The number of heatwave days ranged from 0 to 36 (average, 27.8 days) in Gurye and 22 to 39 (average, 36.5 days) in Sunchang (Figure 5). Unlike the number of heatwave days in Gurye (29) announced by the KMA, spatial differences were observed depending on the census output area. In particular, for a census output area with extensive forest cover, the number of heatwave days was found to be zero. In Sunchang, the differences in the number of heatwave days among the 58 census output areas was small, as all values were ≥30 for most areas. Specifically, the smallest number of heatwave days in Sunchang (22) was different from that of Gurye (0). Heatwave duration is correlated with increasing heat stress, as high temperatures are maintained, and range from 0 to 19 days (average, 12.6) in Gurye, with low differences among areas. In Sunchang, the number duration ranged from 9 to 29 days (average, 21.9), and values were also generally similar among all census output areas. Both the number of heatwave days and the duration showed significant differences in the census output area distribution of Gurye and Sunchang according to the GPRM. The total number of heatwave days in Gurye in 2018, according to the KMA data, was 29; in comparison, the census output area results showed a maximum of 36 days, representing a significant difference (Figure 5).

### 3.3. Heat Exposure in the Pedestrian Environment

In the heat exposure analysis of the pedestrian environment, the distribution at the level of census output areas was analyzed by calculating the shadow pattern, SVF, and mean radiant temperature. Specifically, shadow pattern was calculated every 30 min from immediately after sunrise (06:00) to immediately before sunset (19:30), and the resulting time unit, as well as the daily average shadow pattern (ranging between 0 to 1). As the shadow pattern approached zero, more shadows were generated via the surrounding terrain, vegetation, and buildings over the grid. Comparatively, SVF quantifies the influence of obstacles that obscure the sky, describing complex geometric characteristics and the incidence relationship with solar radiation. An SVF value of 1 occurs on flat ground with no nearby obstacles, whereas a value of 0 is obtained when the sky is completely obscured by nearby terrain, buildings, or vegetation. In the present study, a 5 m resolution SVF was calculated for Gurye and Sunchang according to the methods of Yi et al. [21]. The longwave and shortwave radiation fluxes, as well as the mean radiant temperature (used for final heat vulnerability assessment) were calculated based on the shadow pattern and SVF information. The mean radiant temperature depicts the average temperature of the surrounding surface that exchanges heat with the human body through radiation, indicating the sum of the shortwave and longwave fluxes of the non-uniform surfaces that surround a human body. In Europe, mean radiant temperature is primarily used to assess thermal comfort [27]. 

**Figure 6:**
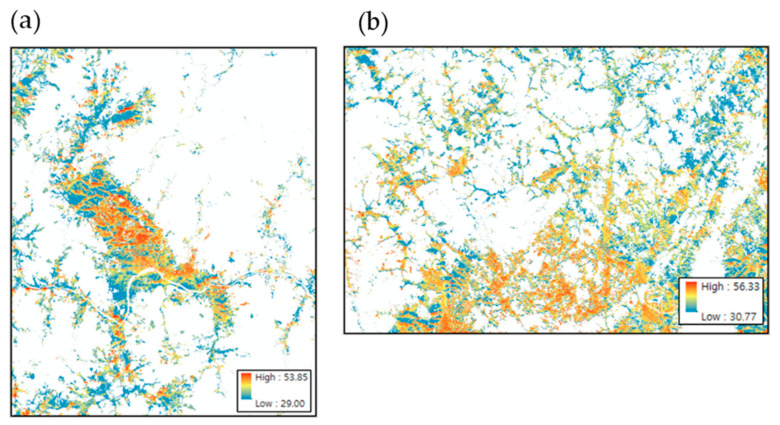
Average daytime Tmrt in urbanized agricultural areas of Gurye (**a**) and Sunchang (**b**).

**Figure 7:**
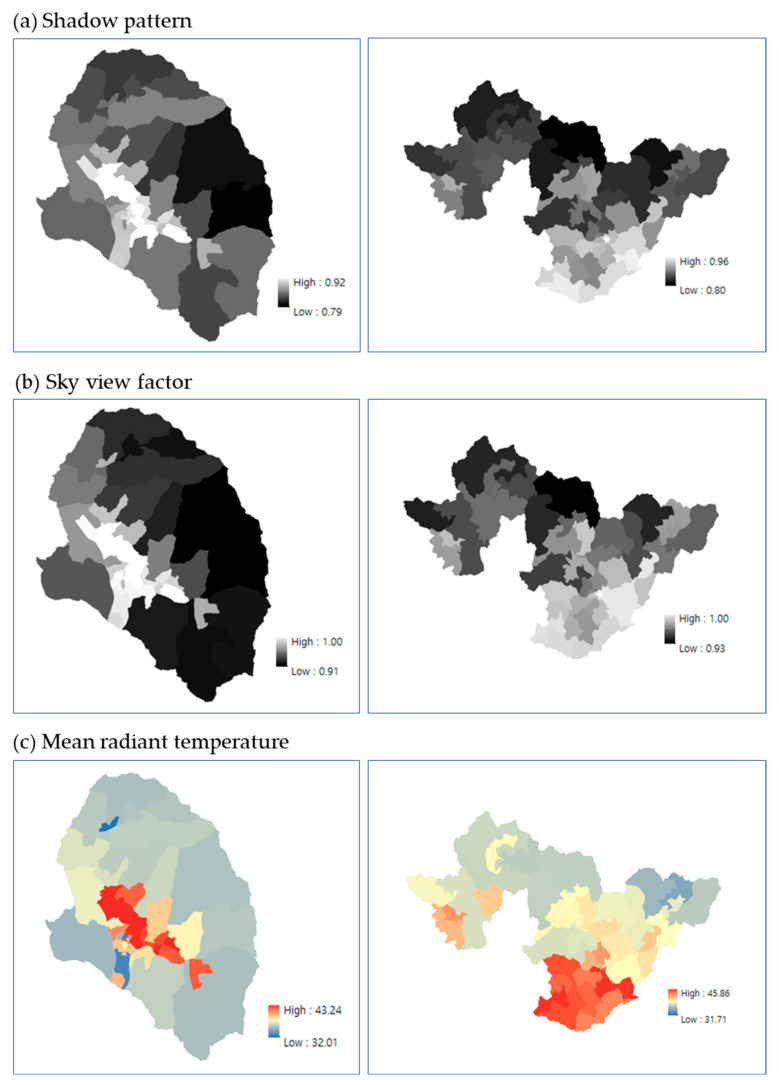
Distribution of shadow patterns, sky view factor, and mean radiant temperature in Gurye and Sunchang.

On 1 August 2018, when the largest number of heatwave-related deaths occurred, the average daytime mean radiant temperature (Tmrt) ranged from 29.0 °C to 53.8 °C (average, 34.1 °C) in Gurye and from 30.8 °C to 56.3 °C (average, 37.3 °C) in Sunchang. The maximum and minimum mean radiant temperatures of Sunchang were similar to those of Gurye; however, the average mean radiant temperature was 3.2 °C higher in Sunchang. From the calculated mean radiant temperature distribution, excluding forested and river areas, only urbanized and agricultural areas (where many people reside) were extracted using a land cover map and compared (Figure 6). Here, the daily mean radiant temperature (average daytime Tmrt) ranged from 29.0 °C to 53.9 °C (average, 35.6 °C) in Gurye and from 30.8 °C to 56.3 °C (average, 39.2 °C) in Sunchang, whereas the mean radiant temperature of Sunchang was found to be 3.6 °C higher than that of Gurye, resulting in a larger difference than the average over the entire area. For the spatial distribution analysis, the shadow pattern, SVF, and mean radiant temperature calculated in SOLWEIG were converted according to census output area using the zonal statistics method (Figure 7).

### 3.4. Spatial Autocorrelation of Heatwave Damage Vulnerability Factors

We present in Table 5 the Moran’s index, *p*-values, and cluster distribution characteristics calculated through the global spatial autocorrelation analysis. A negative value for Moran’s *I* indicates the dispersion of high or low attribute units. The spatial autocorrelation analysis results for each vulnerability factor were found to differ between Gurye and Sunchang. In Gurye, vulnerability factors with a Moran’s index of ≥0.5 included the number of heatwave days, duration of heatwave, number of tropical nights, access to medical institutions, urbanization rate, capacity of the hot shelter, access to the hot shelter, population density, and number of cardiac infarction patients. In Sunchang, shadow pattern, SVF, number of old houses, urbanization rate, capacity of the hot shelter, access to the hot shelter, grade of old houses, average age, cardiac infarction, and stroke met this criterion (Table 5). As for the detailed indicators of heatwave risk, the hazard factors showed a high correlation in Gurye, whereas the vulnerability factors exhibited a high correlation in Sunchang.

**Figure 8:**
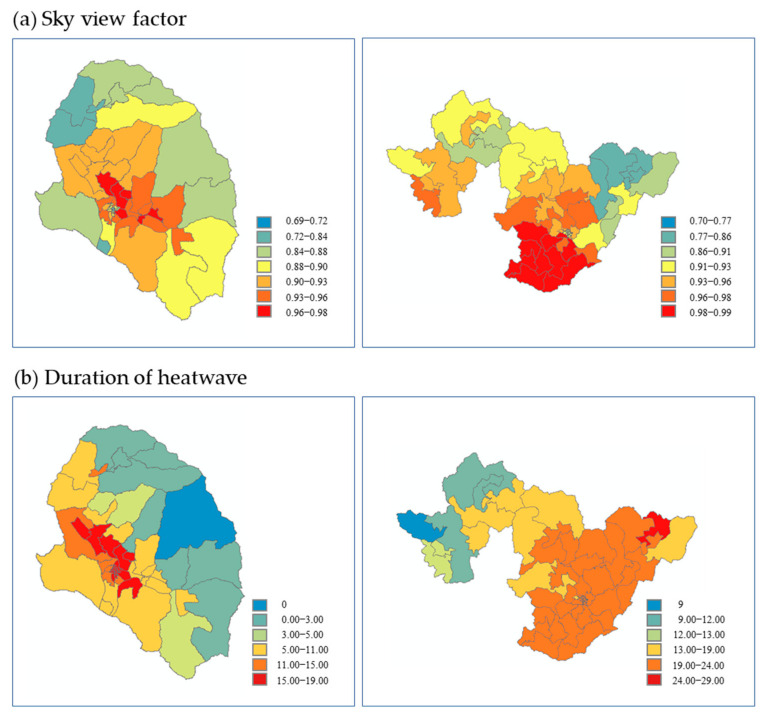
Gurye and Sunchang SVF values and duration of heatwave.

Regarding the hazard factors of heatwave risk corresponding to weather factors, heatwave duration and SVF showed the largest differences between Gurye and Sunchang. While both the duration of heatwave and SVF were significantly different among the census output areas and did not show clustered characteristics that were evident in Gurye, there were marked high-value clusters in Sunchang (Figure 8). Among the hazard factors corresponding to weather factors, SVF and heatwave duration, which showed promising discrimination ability, as expressed through the largest differences between the two regions, are notably not provided by Statistics Korea or any other institutions but were calculated in the present study. Accordingly, it appears that the vulnerability factors of the regions can be distinguished under analyses at the census output area level due to the more detailed and diversified weather factors incorporated compared to previous studies. Specifically, the added factors representing the degree of bodily sensation in the pedestrian environment, which reflect the high-resolution ground surface data and surrounding environmental characteristics, were shown to be the most influential.

In Sunchang, where the customized data set of the NHIS showed a larger number of patients with heat-related illnesses, a total of 11 factors among the heatwave risk vulnerability factors (two of five hazard factors, six of seven vulnerability factors, and three of thirteen exposure factors) showed higher spatial autocorrelation. In Gurye, where a larger number of patients with heat-related illnesses was reported, a total of nine factors among the heatwave risk vulnerability factors (three of five hazard factors, four of seven vulnerability factors, and two of thirteen exposure factors) showed higher spatial autocorrelation (Table 5).

**Figure 9:**
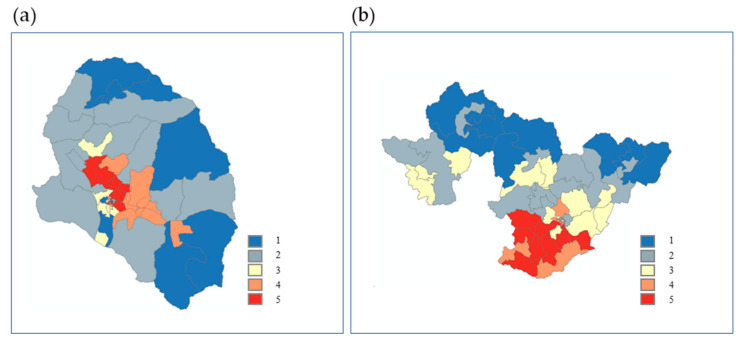
Heatwave risk in (**a**) Gurye and (**b**) Sunchang.

In Sunchang, the shadow pattern and SVF, which represent bodily sensation among the weather factors of the hazard category, were both found to be high. Further, there was a strong correlation between the number and grade of old houses, which are the environmental factors of the vulnerability category. The infant population, elderly population, aging index, and support fee for the elderly were high among the personal factors of the vulnerability category, whereas cardiac infarction and stroke were high among the disease factors. For Gurye and Sunchang, similar vulnerability and exposure factors were represented by MOE (Table 4). It was found, however, that the vulnerability index varies depending on how detailed and diverse the environmental factors are within the vulnerability category and if the personal and disease factors are within the exposure category (i.e., whether factors that represent personal characteristics were created spatially). The heatwave risk maps of Gurye and Sunchang were calculated using the mean radiant temperature and spatial autocorrelation analysis results, which can represent the degree of heatwave risk by reflecting the high-resolution ground surface and surrounding environmental characteristics (Figure 9). These maps were used to identify areas with high heatwave damage risks by reflecting vulnerability factors with high spatial autocorrelation. The applied weight for each vulnerability factor was 0.37 for hazard, 0.27 for vulnerability, and 0.36 for exposure.

The heatwave risk map was plotted using the mean radiant temperature, representing thermal vulnerability, and the value of the vulnerability factor to which weights for each factor were applied. The spatial scale unit is the census output area and the risk grade of the heatwave map is divided into grades 1 to 5, using the Jenks grade calculation method, with “5” indicating the highest risk of heatwave.

Grade 5 risk was mainly distributed in urban areas of Gurye. Among the 50 census output areas, 11 showed grade 5, which corresponds to 3.1% of the total regional area. Comparatively, in Sunchang, seven out of fifty-eight census output areas were identified as grade 5, corresponding to 10.4% of the total area. The portions of census output areas that exhibited grade 5 in Gurye and Sunchang were 22% and 12%, respectively. Sunchang is a larger land area than Gurye, and census output areas with grade 4 were primarily distributed around the grade 5 locations. Although census output areas with grades 1 and 2 were widely distributed in Gurye, grades 1–4 were relatively sporadically distributed in Sunchang. In Gurye, the proportions of grades 4 and 5 were found to comprise 38% (19) of the number of census output areas and 7.2% of the total area. Comparatively, in Sunchang, the proportions of grades 4 and 5 comprised 34.5% (20) of the census output areas (20) and 21.4% of the total area. Accordingly, the proportion of the area with high heatwave risk was approximately three times higher in Sunchang than in Gurye, with the more widely distributed risk area in the former indicating that if residential areas are located in locations with high heatwave risk, the proximate living areas are also likely to exhibit such risk.

### 3.5. Characteristics of Heat-Related Illness in Gurye and Sunchang

The number of heat-related illness patients in 2018, as recorded by the Korea Disease Control and Prevention Agency (KDCA), was sixteen in Gurye and three in Sunchang, more than five times higher in Gurye (Table 6). In Gurye, 14 cases of heat illness occurred outdoors and, among heat illnesses, there were eight cases of T67.0 (heat stroke) in Gurye and no reported heat illnesses in Sunchang. Based on the KDCA patient counting method, it can be seen that more heat-related emergency cases occurred in Gurye than in Sunchang. According to the National Health Insurance Service (NHIS), the number of heat-related illness patients in 2018 was approximately three times more in Sunchang than in Gurye (26 in Gurye and 77 in Sunchang). Among them, nine cases of T67.0 (heat stroke) occurred in Gurye and forty-seven in Sunchang. The number of reported cases is somewhat different than what was reported by the KDCA: the number of cases in Sunchang was higher in the NHIS count, which is the number of heat-related patients who visited hospitals and received treatment. The KDCA data, related to emergencies, are highly related to the occurrence of acute and emergency cases; the NHIS data, related to hospital visits, are data collected when people voluntarily go to the hospital. As the cases are counted differently in the two data sets, it is necessary to select and use the data most suitable for the purpose.

**Figure 10:**
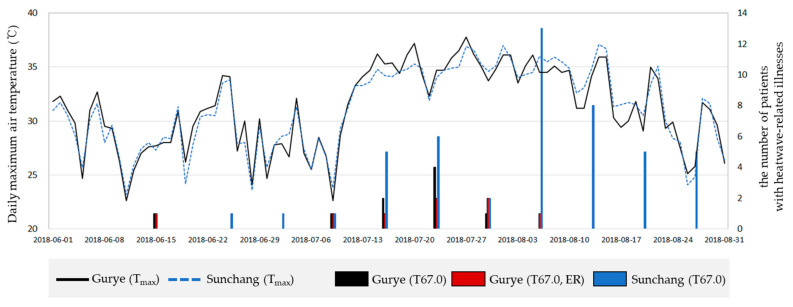
Daily maximum temperature and occurrence of T67.0 cases, as reported by the National Health Insurance Service (NHIS).

The time series change of the daily maximum air temperature and the number of T67.0 cases (summed per week) from 1 June 2018 to 31 August 2018 were plotted (Figure 10). The number of patients began to increase in both regions from 6 July 2018, when the daily maximum temperature increased rapidly; in Gurye, cases occurred until August 3. Heat illness outbreaks continued until August 31, while the temperature persisted at approximately 35 °C.

The cause of this continuous occurrence to late summer can be explained in relation to the results analyzed in Section 3.4. As a result of calculating the heatwave risk map, 12 out of 50 counted by census output area in Gurye showed a heatwave risk level of 5, accounting for approximately 3.4% of the total area of the administrative district. In Sunchang, seven out of fifty-eight counted by census output area showed a heatwave risk level of 5 and it was analyzed that they accounted for approximately 10.4% of the total area of the administrative district. Although the number of grade 5 counts in the census output area in Sunchang is smaller than in Gurye, the area (spatially exposed area to heatwave risk) is approximately three times wider.

In the hazard factors (sky view factor and mean radiant temperature related to physical residential environment among meteorological factors) and vulnerability factors, Sunchang clearly showed clustered characteristics with high values. In Sunchang, which has more areas and elements representing grade 5 heatwave risk, the number of patients with heat illness (T67.x) was three times higher and those with severe T67.0 were five times higher than the corresponding numbers in Gurye. On the basis of these results, it can be concluded that physical factors can affect illness occurrence.

## 4. Conclusions

In the present study, the detailed causes of heatwave vulnerability were analyzed at the level of census output areas in Gurye, Jeollanam-do, and Sunchang, Jeollabuk-do, South Korea, as they had previously been selected as study areas for assessing the severe heatwave-induced damages during the summer of 2018. Further, the causes of the differences between the two counties were identified through spatial autocorrelation analyses. Weather, environmental, personal, and disease factors at the level of census output areas were constructed as heatwave damage vulnerability factors, and factors reflecting the pedestrian environment and personal characteristics of residents were calculated to increase the detail and diversity of the public data used.

Spatial autocorrelation analyses revealed that hazard factors were highly correlated with heatwave risk in Gurye, whereas vulnerability factors exhibited a high correlation in Sunchang. The duration of heatwave and SVF that exhibited the largest difference in the spatial autocorrelation analyses comprised the variables distinct to this study. Accordingly, it was concluded here that the vulnerability factors of the regions were distinguished when heatwave vulnerability was analyzed at the census output area level due to the detailed and diversified weather factors incorporated here that represent the degree of bodily sensation in the pedestrian environment. Further, this also reflects the importance of incorporating high-resolution ground surface and surrounding environmental characteristics in such analyses.

The enhanced method of analyzing causes of heatwave vulnerability at the census output area level proposed here, as well as the corresponding results, can inform more detailed heatwave response policies for local governments. In addition, because the number of patients with heat-related illnesses differed depending on the data for each region, it is necessary to analyze and interpret the detailed causes of heatwave vulnerability when the heatwave damage statuses at the city, county, and district levels are analyzed. If a comparative analysis is performed by drawing up detailed heatwave risk maps with various types of heatwaves, it is thought that the characteristics of heatwave damage occurrence according to the regionality and heatwave type can be identified.

Future analyses of the causes of heatwave damage should contain more detailed, and a wider variety of factors that represent even the personal characteristics of local community members, rather than solely the temperature-dependent risk levels provided by the Korea Meteorological Administration (KMA) used to create heatwave alerts and warnings. In particular, it is necessary to develop various heatwave vulnerability assessment techniques by adding an analysis that reflects multiple factors, even though it is difficult to quantitatively assess some social factors at the individual level.

‘Adaptive capacity’, such as heat mitigation and heat adaptation in the target area, was not applied to the heatwave risk map prepared in this study. The heatwave risk map of more diverse regions and the analysis of heatwave response policies by local government should be performed together, and it is thought that the additional application of ‘socioeconomic’ factors such as income, education, and acclimatization will be necessary.

## Figures and Tables

**Table 1:** Statistical data on health damages in heatwaves.

Data	Data Provider	Explanation
Heat-related illnesses surveillance	Korea Disease Control and Prevention Agency (KDCA)	Information on patients who visited the emergency room due to heat-related illnesses and mortalities (T67)
Customized research data	National Health Insurance Service (NHIS)	Health information prepared based on insurance premium claim data submitted by all medical institutions

**Table 2:** Main symptoms and disease codes of heat-related diseases (KCD–8th Revision).

Code	Classification	Major Symptoms
T67.0	Heatstroke and sunstroke	Central nerve dysfunction
T67.1	Heat syncope	Fainting, Dizziness
T67.2	Heat cramp	Muscle spasm
T67.3 T67.4 T67.5	Heat exhaustion, anhydrotic	Raising body temperature, Excessive sweating, Sense of helplessness, Vomiting
T67.7	Heat edema	Edema
T67.8 T67.9	Other effects of heat and light	

**Table 3:** Heatwave vulnerability factors.

	Factors Determining Vulnerability to Heatwave Damage
Weather factors	Heatwave days Duration of heatwave Tropical night days Shadow pattern value Sky view factor
Environmental factors	Grade of old houses Number of old houses Number of nursing homes
Personal factors	Infant population Elderly population Population density Average age Aging index Support fee for the elderly Agriculture population
Disease factors	The number of patients with underlying diseases (hypertensive, diabetes, cancer, cardiac infarction, stroke)

**Table 4:** Detailed indicators of the number of people with thermal diseases, and the risk of heatwaves in Gurye and Sunchang (2018).

Patients with Heat-Related Illness			Exposure			Vulnerability		
	Gurye	Sunchang		Gurye	Sunchang		Gurye	Sunchang
KDCA	16	3	Ratio of elderly population	31.4%	31.8%	Urbanized area ratio	2.46%	2.04%
NHSI	27	152	Ratio of infants and toddlers	2.0%	3.2%	Green area ratio	74.41%	90.24%
			Ratio of elderly living alone	7.4%	8.6%	Water area ratio	2.62%	2.52%
**Hazard**			Ratio basic livelihood recipients	5.0%	3.5%	Gross regional domestic product (GRDP)	553,644 (million won)	720,399 (million won)
	Gurye	Sunchang						
TMAX	30.0 °C	31.6 °C	Total population	27,117	29,209	Financial independence (before the reorganization)	18.5%	14.8%
Heatwave days	29	40	Female population	13,191	14,156	Financial independence (after the reorganization)	6.3%	6.3%
Tropical night days	2	4	Male population	13,926	15,053	Ratio of people covered by health insurance	95.7%	96.4%
Relative humidity	82.3%	75.6%	Population density	61.18 people·km^−2^	58.9 people·km^−2^	Number of shelters in the heat	176	159

**Table 5:** Moran’s index for each heatwave vulnerability factor in Gurye and Sunchang (Moran’s *I* > 0.5 written in bold).

Hazard			Exposure		
	*Gurye*	*Sunchang*		*Gurye*	*Sunchang*
Heatwave days	0.526 (*p* < 0.000001)	0.402 (*p* < 0.000001)	Infant population	0.192 (*p* < 0.000001)	0.427 (*p* < 0.000001)
	Clustered	Clustered		Clustered	Clustered
Duration of heatwave	0.591 (*p* < 0.000001)	0.017 (*p* = 0.454450)	Elderly population	0.242 (*p* < 0.000001)	0.475 (*p* < 0.000001)
	Clustered	Random		Clustered	Clustered
Tropical night days	0.704 (*p* < 0.000001)	0.437 (*p* < 0.000001)	Population density	0.601 (*p* < 0.000001)	0.265 (*p* = 0.001243)
	Clustered	Clustered		Clustered	Clustered
Shadow value	0.383 (*p* < 0.000001)	0.739 (*p* < 0.000001)	Average age	0.167 (*p* < 0.000001)	0.580 (*p* < 0.000001)
	Clustered	Clustered		Clustered	Clustered
Sky view factor	0.036 (*p* = 0.236856)	0.703 (*p* < 0.000001)	Aging index	0.254 (*p* < 0.000001)	0.457 (*p* < 0.000001)
	Random	Clustered		Clustered	Clustered
Vulnerability			Support fee for the elderly	0.189 (*p* < 0.000001)	0.493 (*p* < 0.000001)
Number of old houses	0.008 (*p* = 0.556152)	0.501 (*p* < 0.000001)		Clustered	Clustered
	Random	Clustered	Agriculture population	0.057 (*p* = 0.401805)	0.050 (*p* = 0.392016)
Number of local health and medical institutions	0.077 (*p* < 0.000001)	0.044 (*p* = 0.467206)			
	Clustered	Random		Random	Random
Access to medical institutions	0.696 (*p* < 0.000001)	1.155 (*p* < 0.000001)	Hypertensive	0.064 (*p* < 0.000001)	0.177 (*p* < 0.000001)
	Clustered	Clustered		Clustered	Clustered
Grade of old houses	0.180 (*p* < 0.000001)	0.738 (*p* < 0.000001)	Diabetes	−0.035 (*p* = 0.756316)	−0.064 (*p* = 0.594331)
	Clustered	Clustered		Random	Random
Urbanization rate	0.992 (*p* < 0.000001)	1.448 (*p* < 0.000001)	Hyperlipidemia	0.008 (*p* = 0.550881)	−0.015 (*p* = 0.974844)
	Clustered	Clustered		Random	Random
Capacity of the hot shelter	0.584 (*p* < 0.000001)	0.790 (*p* < 0.000001)	Cancer	0.102 (*p* < 0.000001)	0.359 (*p* < 0.000001)
	Clustered	Clustered		Clustered	Clustered
Access to the hot shelter	0.557 (*p* < 0.000001)	1.080 (*p* < 0.000001)	Cardiac infarction	0.753 (*p* < 0.000001)	1.104 (*p* < 0.000001)
	Clustered	Clustered		Clustered	Clustered
			Stroke	0.230 (*p* < 0.000001)	0.651 (*p* < 0.000001)
				Clustered	Clustered

**Table 6:** Number of patients by reporting institution (KDCA vs. NHIS) and heat-related illness code (T67.x) in Gurye and Sunchang (2018).

Study Area	KDCA T67.0	NHIS T67.0	KDCA T67.1	NHIS T67.1	KDCA T67.2	NHIS T67.2	KDCA T67.3~5	NHIS T67.3~5	KDCA T67.3~5	NHIS T67.3~5	KDCA SUM	NHIS SUM
Gurye	8	9	0	0	0	1	4	9	4	7	16	26
Sunchang	0	47	1	1	1	1	1	11	0	17	3	77
